# From Scar to Function: Plastic Surgery in the Management of an Extreme Hand Contracture in a Child

**DOI:** 10.7759/cureus.92121

**Published:** 2025-09-12

**Authors:** Maria Klimeczek-Chrapusta, Anna Chrapusta

**Affiliations:** 1 Faculty of Medicine, Jagiellonian University Medical College, Kraków, POL; 2 Malopolska Burn and Plastic Surgery Center, Ludwik Rydygier Memorial Hospital, Kraków, POL

**Keywords:** burn reconstruction, contraction, hand surgery, pediatric plastic surgery, z-plasty

## Abstract

Children are more prone to develop post-burn contractures than adults, even when optimal initial burn care is provided. In severe cases, such contractures may lead to significant deformities and functional impairments. We present a case of a pediatric patient referred to a plastic surgeon at the age of two by his parents, a few months after sustaining third-degree burns to both hands. Although the burns had healed, extensive scarring resulted in severe contractures, leaving the child’s hands tightly clenched and preventing him from extending, flexing, or moving his fingers, thereby severely impairing hand function. A multi-stage surgical plan was established to release the contractures and restore hand function. Over a six-year period, five reconstructive procedures were performed, two on the left hand and three on the right. The surgical strategy incorporated full-thickness skin grafts (FTSGs), multiple Z-plasties, dorsal pentagonal island flaps, Z-plasties, and the use of Kirschner wires to maintain finger extension following passive joint release and contracture correction. This staged approach resulted in the successful restoration of hand functionality and an aesthetically favorable outcome. The postoperative course was uneventful, with minimal pain and no complications. Beyond the physical reconstruction, the regained hand function significantly contributed to the child’s developmental progress, enhancing autonomy, promoting social interaction, and markedly improving overall quality of life.

## Introduction

Palmar contact burns represent a frequent injury in children, primarily attributable to their high levels of physical activity and limited hazard awareness [1]. Moreover, the inherently thinner dermal layers and delayed withdrawal reflexes characteristic of pediatric patients increase the likelihood of sustaining deeper thermal injuries. The development of post-burn contractures is multifactorial, with contributing elements including partial or full-thickness skin loss and the disparity in growth rates between scar tissue and unaffected skin during the child’s development. Notably, the duration of wound healing is a critical determinant: burns that fail to re-epithelialize within three weeks are significantly more prone to hypertrophic scarring and the formation of contractures, often necessitating subsequent reconstructive intervention [1-4].

Conservative management of hand scars after deep burns typically involves compression therapy, provided that sewing and wearing a custom-made glove are feasible. Additional non-surgical approaches include physiotherapy, occupational therapy, and active exercises to restore function. In older, cooperative children, dynamic splinting may also be considered. Scar-reducing gels such as Contractubex or Cepan are commonly applied with massage several times daily to relieve tension and support recovery. Nevertheless, in some cases, conservative therapy is insufficient to restore optimal hand function, making surgical intervention necessary.

This case report presents a rare instance of severe post-burn contracture resulting in complete loss of hand function. We further detail the reconstructive surgical approach undertaken, which yielded a favorable functional and aesthetic outcome.

## Case presentation

**Figure 1:**
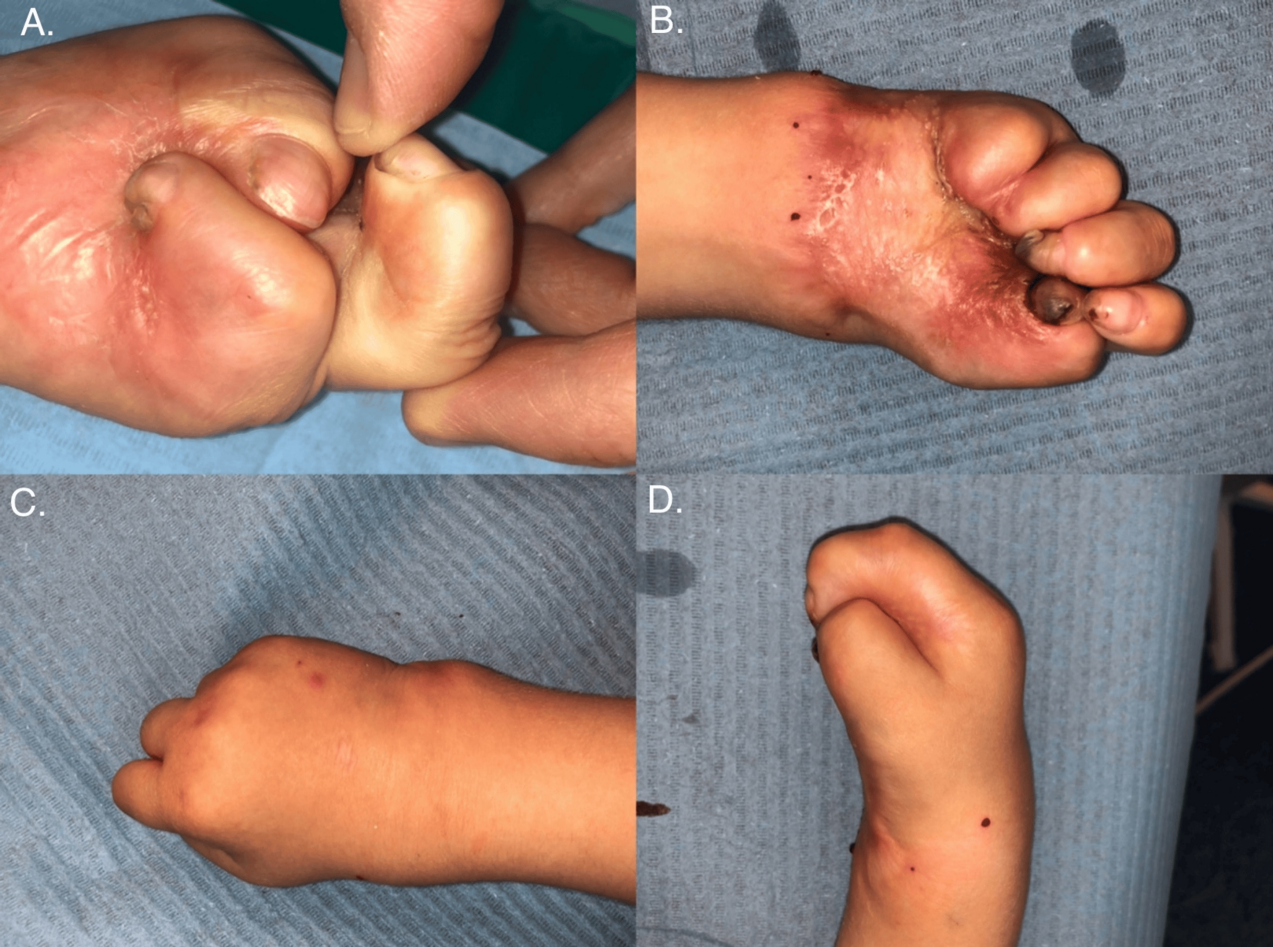
Contracture of the left and right hands before the first surgery

We report the case of a two-year-old boy referred to a plastic surgery clinic with severe flexion contractures affecting the palmar aspects of both hands, leading to complete loss of hand mobility and secondary total syndactyly. The child had previously sustained third-degree thermal burns to both hands following contact with hot ashes. Initial management was carried out at another hospital, where amputation of the distal phalanges of the fourth and fifth digits of the right hand was performed. However, likely due to prolonged wound healing and suboptimal therapeutic interventions, the patient developed extensive contractures during the recovery process (Figure 1). Prior treatment attempts, including laser therapy at an outside facility, failed to halt the progression of the deformities.

**Figure 2:**
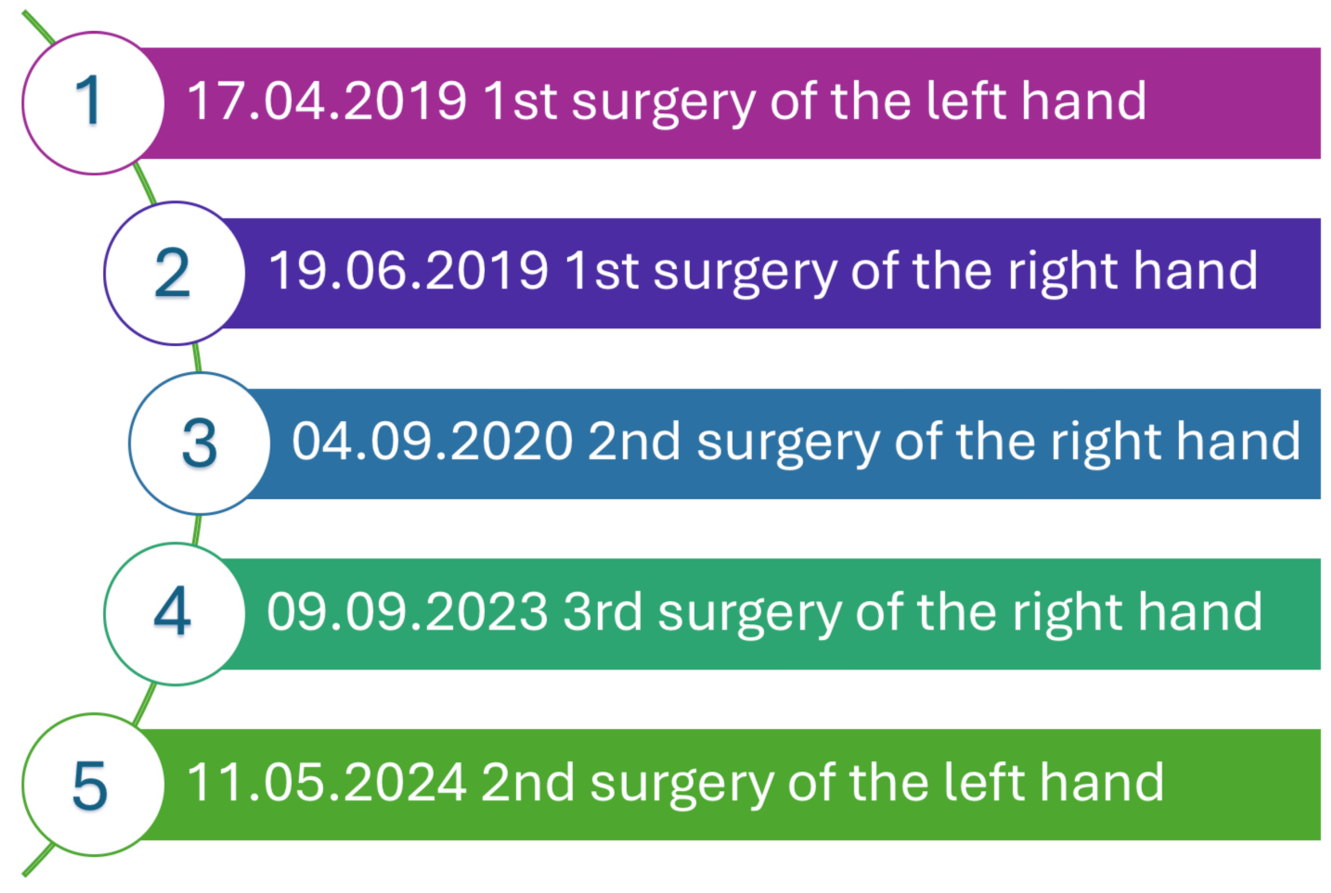
Graph representing the surgeries performed in order

During the preoperative consultation at the clinic, a multi-stage surgical treatment plan was established (Figure 2). At presentation, the child exhibited complete dysfunction of both hands. The left hand was prioritized for the first intervention due to the less advanced degree of contracture and the absence of phalangeal loss, which offered a more favorable prognosis for early functional recovery.

**Figure 3:**
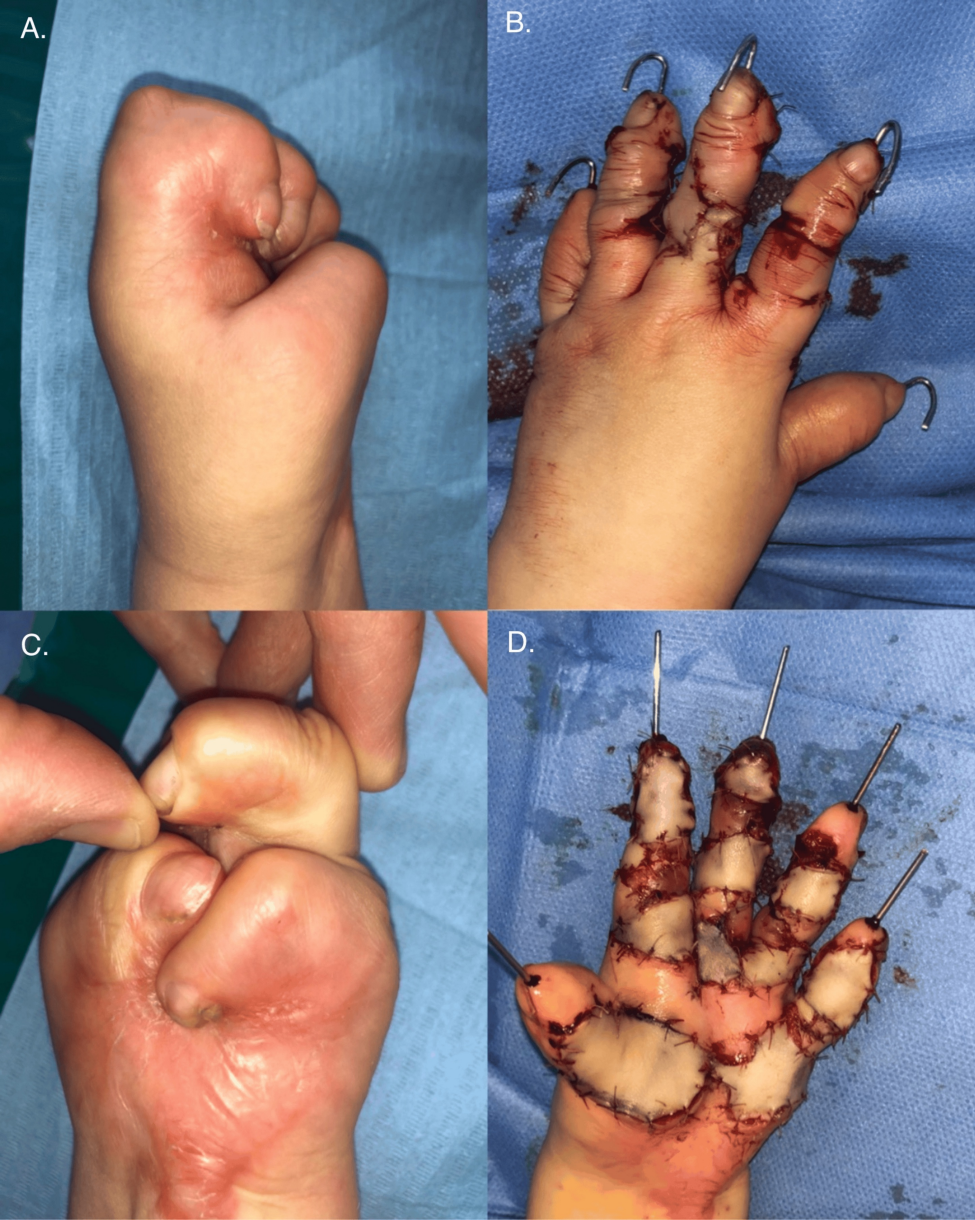
Results of the first surgery on the left hand

The initial surgery on the left hand was performed in April 2019. Surgical release of the palmar contracture was achieved by excising the metacarpal scar between the first and fifth rays, separating digits II-V, and performing multiple Z-plasties along the palmar surfaces of the fingers. The resulting soft tissue defects were covered with full-thickness skin grafts (FTSGs) harvested from the inguinal region (Figure 3). Temporary stabilization of all fingers with Kirschner wires was necessary to maintain extension and facilitate controlled healing. The donor site was closed primarily.

**Figure 4:**
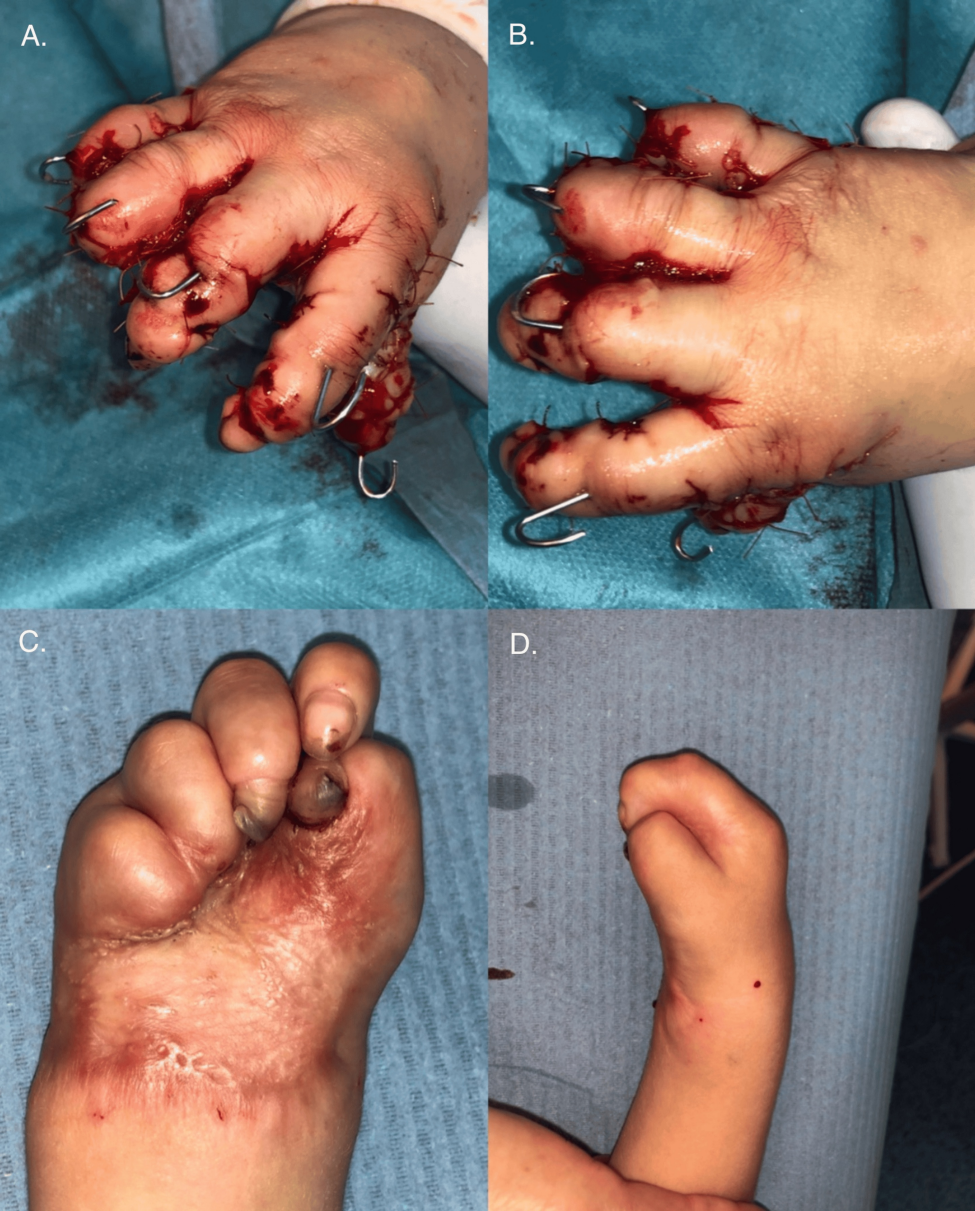
First surgery of the right hand

The first surgery on the right hand was performed two months later. In this hand, digits II-V were entirely fused with one another and with the underlying metacarpals. The surgical approach focused on releasing the extensive adhesions and contractures to enable finger extension. This involved careful dissection to free the neurovascular bundles from surrounding scar tissue, followed by stabilization of the digits in an extended position using Kirschner wires. Where anatomically feasible, Z-plasty was employed to enhance skin mobility and minimize tension. Residual skin defects were reconstructed with full-thickness skin grafts harvested from the inguinal region, opposite to the donor site used during the first procedure (Figure 4).

**Figure 5:**
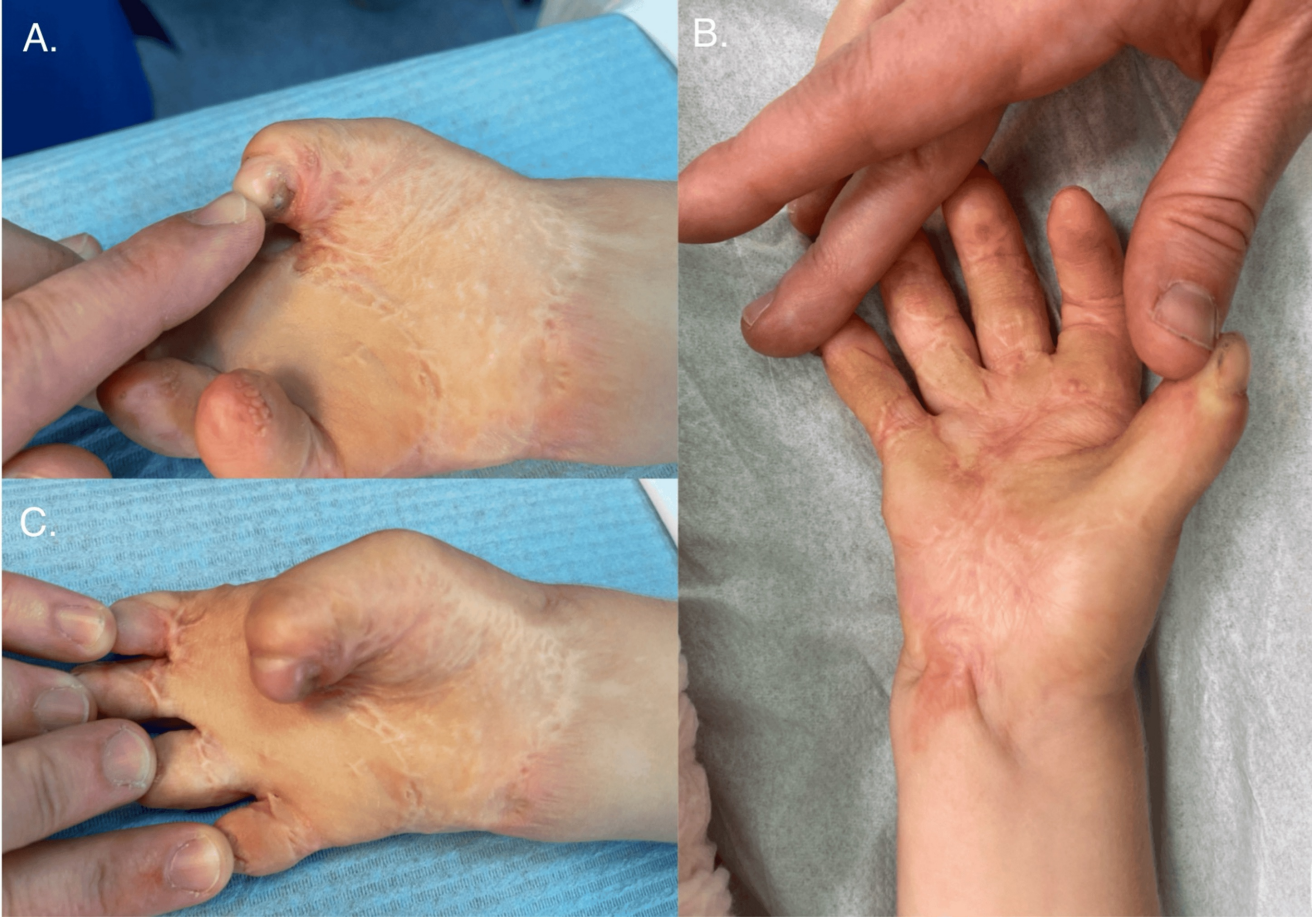
Before the second surgery of the right hand (A and C) and after the second surgery of the right hand (B)

In April 2020, the second surgery on the right hand was performed, employing a combination of reconstructive techniques. Excision of the contracture scar allowed for the release of the thumb. The first interdigital web space was deepened using a Z-plasty, while a dorsal pentagonal island flap was mobilized to facilitate thumb abduction. Additionally, excision of the contracture at the base of the thumb in the metacarpal region further improved thumb extension and abduction. To enhance mobility in the second and third web spaces, a combination of a dorsal pentagonal island flap and butterfly plasty was utilized. Full-thickness skin grafts (FTSGs) harvested from the inguinal region were used to cover the resulting soft tissue defects. The fingers were stabilized with Kirschner wires, and the hand was immobilized in a plaster dressing (Figure 5).

**Figure 6:**
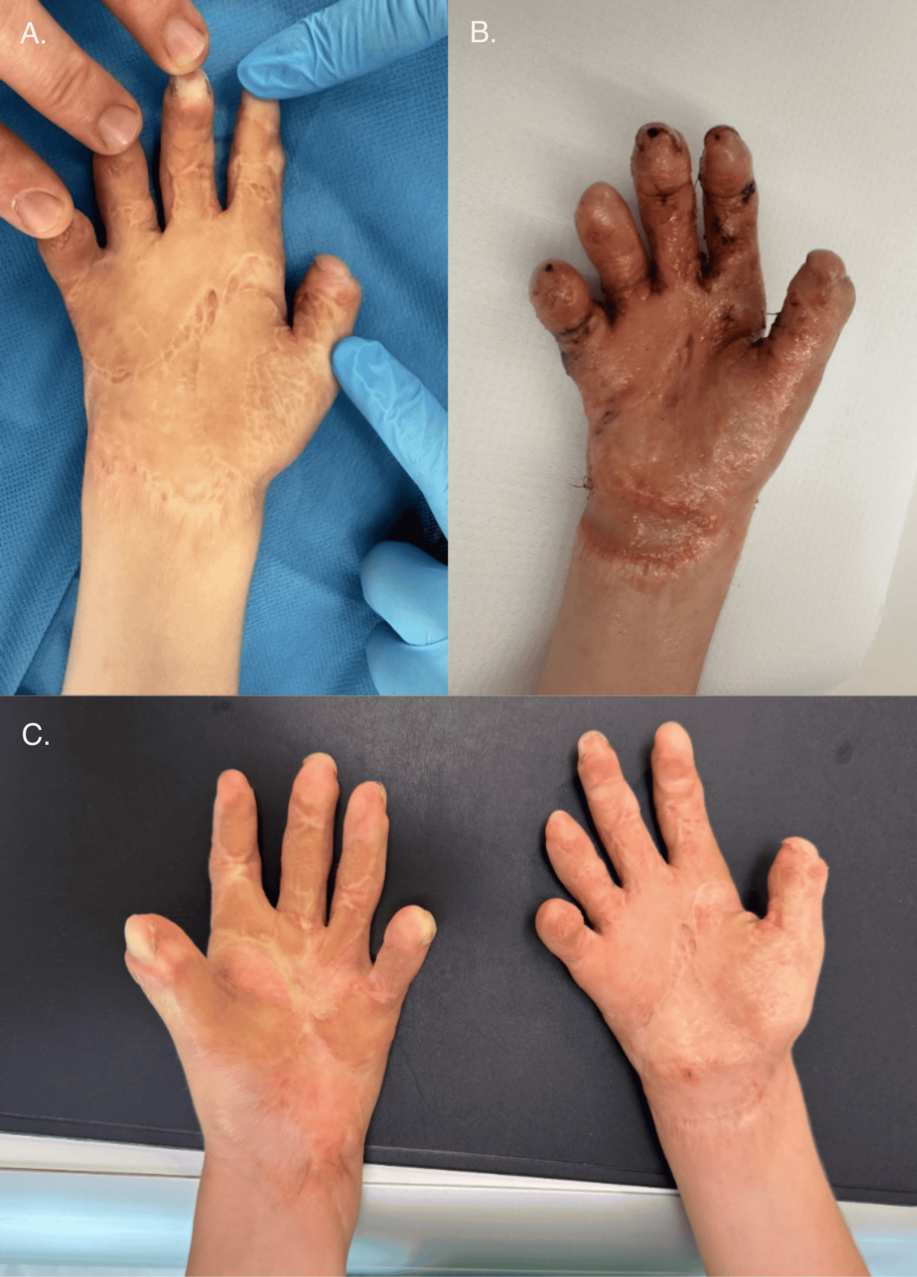
Right hand before and after the third surgery (top) and both hands before the last surgery of the left hand (bottom)

Three years later, in September 2023, a third surgery on the right hand was done. The procedure aimed to improve the range of motion of the thenar eminence and digits I-II through multiple Z-plasties. Residual contractures of the distal and proximal interphalangeal joints of digits II, III, and V were also addressed with Z-plasty techniques (Figure 6).

**Figure 7:**
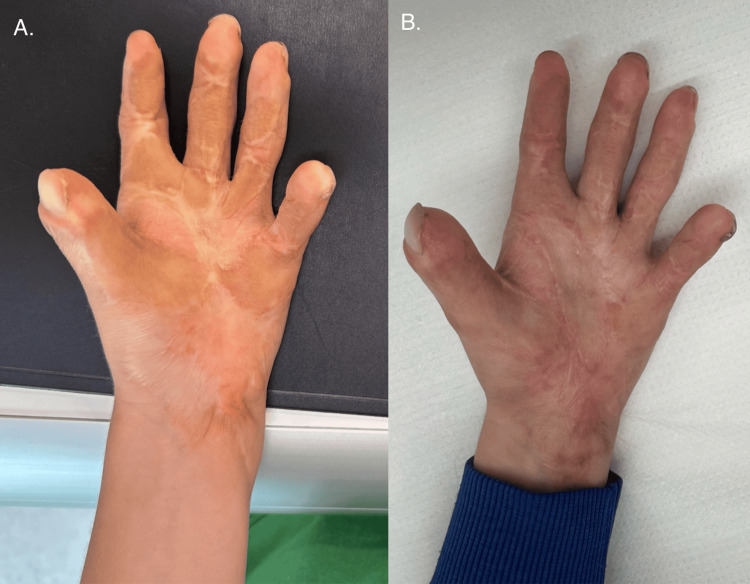
Left hand: before the surgery (left) and after the last surgery (right)

In May 2024, the final surgery was performed on the left hand. A single Z-plasty was carried out to release the wrist contracture, while a double Z-plasty was used to correct the contracture of the first finger. An additional single Z-plasty was applied to the second finger. To further release residual contractures, Z-plasties were performed on the radial side of the base of the fourth finger and the ulnar side of the proximal phalanx of the fifth finger. Temporary Kirschner wires were inserted into the metacarpophalangeal (MCP) joints of the first and fifth fingers to provide stabilization (Figure 7).

**Figure 8:**
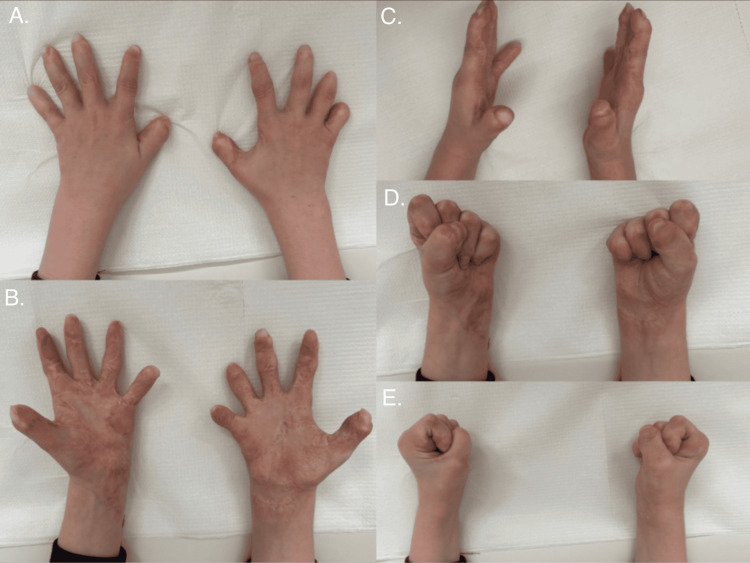
Final results of the treatment process

**Figure 9:**
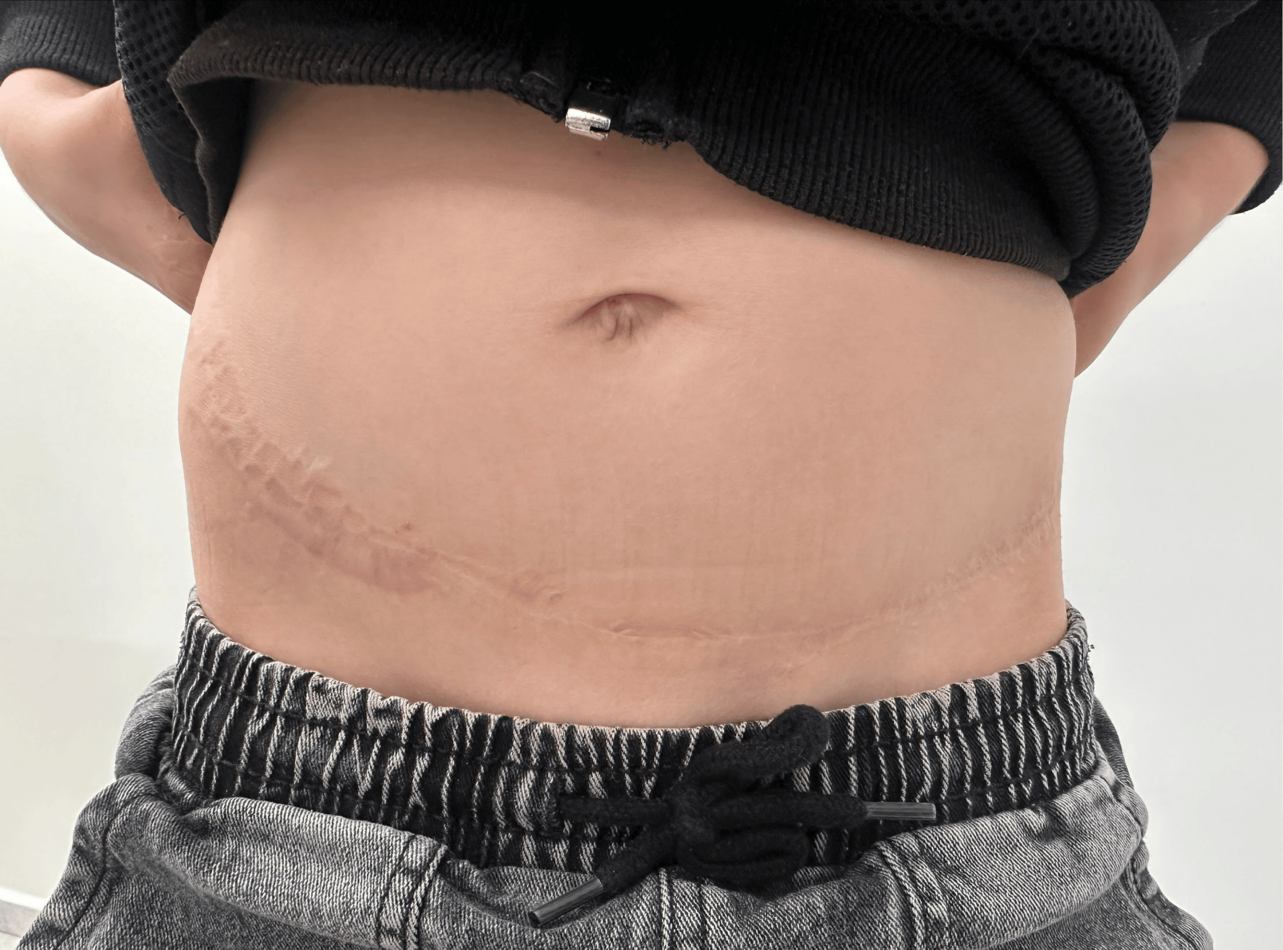
Healed donor sites As evident in the photo, although the scars from graft harvesting are noticeable, they can be easily concealed by clothing.

The final outcome of the multi-stage treatment is illustrated in Figure 8, demonstrating a near-complete restoration of hand mobility. As evident from the images, the patient regained functional use of both hands. Figure 9 presents the fully healed donor sites.

Additionally, the recovery period between the surgeries included physiotherapy. Following the first operation on the left hand and the second on the right, the child was fitted with compression gloves, which were removed only for massage. After each procedure, the parents carried out scar massage five to six times daily using Contractubex gel and a lubricating ointment. These sessions also incorporated finger flexion and extension exercises, with particular attention to the web spaces.

## Discussion

We present a case report of successful contracture release in a child. The management of post-burn hand contractures in toddlers represents a significant challenge in pediatric plastic and reconstructive surgery. In addition to selecting the appropriate surgical technique, clinicians must consider the ongoing impact of growth, as contractures tend to worsen proportionally with the child’s development [1-5].

Barret et al. identified wound healing time as the most critical factor influencing contracture formation [2]. Specifically, palmar burns that required more than three weeks to heal were consistently associated with hypertrophic scarring and contractures, irrespective of burn depth or type. These findings have been supported by studies from Deitch et al. [5] and Grossova et al. [1], highlighting the importance of early and appropriate management of the primary injury.

Liang et al. advocated for the use of full-thickness skin grafts (FTSGs) over split-thickness skin grafts (STSGs) in pediatric patients, citing improved functional outcomes in hand development [3]. Our clinical experience, as well as the outcome of the presented case, support this recommendation. While a systematic review by Prasetyono et al. [6] found no definitive superiority of STSGs over FTSGs, authors included in the review, such as Chandrasegaram and Harvey [7] and Chan et al. [8], endorsed the use of FTSGs due to their superior scar pliability and their appropriateness for treating palmar burns that extend into the digits.

In our case, we propose that reconstruction of the interdigital web spaces should ideally be performed using a dorsal skin flap or, when appropriate, a dorsally pedicled scar. The commissures in this patient were not reconstructed using skin grafts. When feasible, Z-plasty should be used to release digital contractures, as it facilitates continued finger growth while preserving the anatomical oval contour of the digits. To cover residual tissue defects, FTSGs remain the preferred grafting material due to their favorable long-term aesthetic and functional characteristics [2,3].

One of the concerns commonly cited regarding FTSGs is the potential for increased hair growth at the graft site. However, by harvesting the graft from the inguinal region, this issue can be largely avoided due to the low density of hair follicles and the light coloration of the skin in this area [9]. We propose that the inguinal region (Figure 9) is a superior donor site, especially if chosen slightly higher than usual, similar to the lower abdominal region recommended by Liang et al. [3], as it has fewer hair follicles and results in less visible scarring, which can be more easily concealed with clothes.

Although this case report primarily focuses on the surgical management of severe palmar contracture, peri- and postoperative physiotherapy also played a crucial role. The child wore custom-made compression gloves, removed only for massage, while the parents performed scar massage five to six times daily with gel and ointment, combined with finger flexion-extension exercises emphasizing the web spaces. Prasetyono and Caroline reported a similar case with less severe contracture, where poor compliance with splinting and exercises led to recurrence within a year [10]. To address this, they developed a customized two-sided splint, underscoring both the importance of parental involvement and the value of tailored splinting in pediatric burn contracture management.

A review of volar hand burns by Yuen et al. identified only four articles describing the use of combined Z-plasties [11], while most reports focused on management with skin grafts alone (48 articles) or skin substitutes such as Integra combined with grafts (17 articles). In our case, we successfully restored function in both hands using Z-plasties and full-thickness skin grafts (FTSGs), a technique rarely reported in the literature according to the cited review [11].

## Conclusions

This case highlights a rare and extreme presentation of pediatric palmar burn, marked by a complex healing process that culminated in complete secondary syndactyly of all fingers and severe functional impairment. The favorable outcome achieved underscores the importance of timely intervention, tailored management strategies, and effective postoperative care in preventing long-term disability. The multi-stage surgical approach described may offer valuable guidance for clinicians managing similarly challenging cases, emphasizing the critical role of comprehensive rehabilitation in optimizing recovery and preserving hand function.
